# One-Pot Interference-Based Colorimetric Detection of Melamine in Raw Milk via Green Tea-Modified Silver Nanostructures

**DOI:** 10.1021/acsomega.3c09516

**Published:** 2024-05-09

**Authors:** Upama Das, Rajib Biswas, Nirmal Mazumder

**Affiliations:** † Applied Optics and Photonics Research Laboratory, Department of Physics, Tezpur University, Tezpur 784028, Assam, India; ‡ Department of Biophysics, Manipal School of Life Sciences, Manipal Academy of Higher Education, Manipal 576104, Karnataka, India

## Abstract

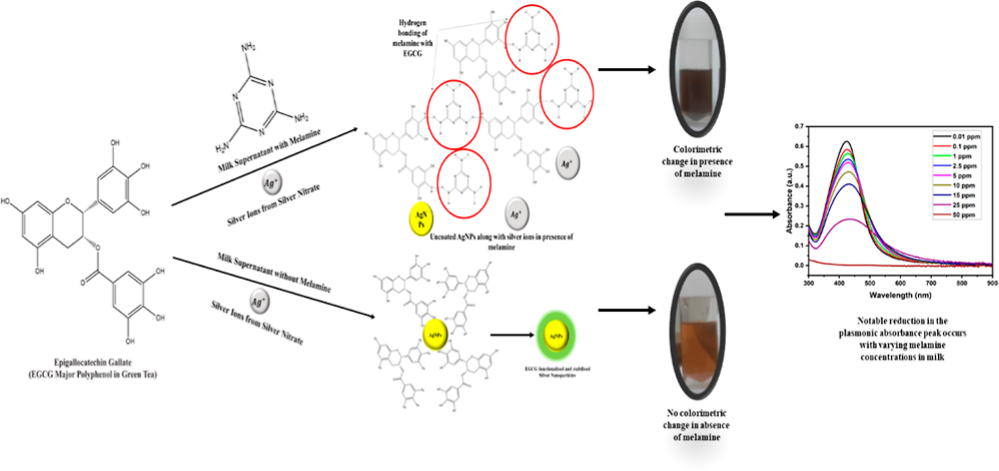

Detection of melamine has proven to be a challenge, requiring the use of complex analytical techniques. This study introduces an innovative, straightforward one-pot technique for qualitative assessment of the milk adulterant melamine. Originally, silver nanoparticles (AgNPs) were synthesized by utilizing green tea extract, which acted as both a reducing and sensing element. The synthesized AgNPs were characterized using UV–vis spectroscopy, X-ray diffraction, zeta potential, transmission electron microscopy, field emission scanning electron microscopy, and Fourier transform infrared spectroscopy. Melamine, rich in –NH_2_ groups, interacts with the biopolyphenols of green tea extract through hydrogen bonding. This interaction inhibits the formation of nanoparticles, resulting in a noticeable colorimetric response. The data obtained were confirmed by a standard UV–vis spectrophotometer and validated by the high-performance liquid chromatography technique. The limit of detection achieved by this scheme was quite low, falling below the permissible levels recommended by government bodies, e.g., the Food Safety and Standards Authority of India (FSSAI).

## Introduction

Milk adulteration has been a pervasive issue plaguing society for centuries. It means addition of undesirable foreign substances to milk, which thereby degrades its quality and renders it unfit for consumption.^1^ Various categories of adulterants are added to milk, which increases the amount of nonprotein nitrogen content in milk through the introduction of toxic nitrogen-rich compounds like melamine and urea.^2^ Meanwhile, others are intended to extend its shelf life, and these may include toxic and carcinogenic substances like salicylic acid, formalin, and hydrogen peroxide.^3^ Adulterants like milk from different species or even water are added to increase milk quantity,^1^ while some other chemicals such as ammonium sulfate are introduced to milk to maintain higher density.^4^ But all of these adulterants are highly toxic and also possess carcinogenic effects.

However, among the various types of adulterants, melamine emerges as one of the most commonly used highly toxic milk adulterants. It comprises a significantly high proportion of nitrogen, extending to 66% of its mass.^5^ It is used in various industrial applications for preparation of plastics, kitchenware, cookware, resins, laminates, plywood, floor tiles, electrical components, etc.^6^ Due to its extreme hazardous nature, its use in food applications has been completely banned by governmental food regulatory bodies.^7^

Dealers maliciously add water to milk to increase its quantity, diluting the net protein content. Some tests such as the Kjeldahl and Dumas tests were employed to assess the net protein amount in milk by estimating its total nitrogen content.^8^ Consequently, the dealers intentionally introduce melamine into milk to augment its nitrogen levels,— thereby compensating for the decrease in protein content of milk which occurs when milk is diluted. The artificial elevation of nitrogen content falsely suggests a higher content of protein in the milk.^9^

Meanwhile, metal nanoparticles have emerged as a captivating tool applied in varied colorimetric-based sensing assays due to a unique phenomenon known as localized surface plasmon resonance (LSPR), e.g., identification of adulterants in milk,^10^ sensing of pesticides,^11,12^ determination of heavy metal content in water and milk,^13^ and more. Silver and gold nanoparticles (AgNPs and AuNPs), primarily, are harnessed for designing these colorimetric assays owing to their exceptional plasmonic properties in the visible spectrum.^14^ In comparison to gold nanoparticles, the use of silver nanoparticles grabs greater attention for colorimetric-based applications, primarily owing to their exceptional excitation coefficients and cost-effectiveness compared with that of the former. This feature enhances their use as colorimetric sensors.^15^ However, it is important to note that their highly unstable nature is a drawback associated with the use of silver nanoparticles because of their high oxidizing properties. However, this challenge can be mitigated by using a suitable functionalizing or capping agent which serves to enhance their stability.^16^

The current literature includes numerous chromatographic techniques for detecting melamine in milk, but these methods are highly sophisticated and necessitate intricate pretreatment procedures prior to detection.^17−19^ Some colorimetric approaches are also reported in literature; however, these methods entail a long pretreatment process and an extended sensing procedure, thereby demanding a substantial amount of chemicals for effective detection of the adulterant.^8,20−24^

Unlike existing methods, our approach introduces an innovative and straightforward method to qualitatively assess the melamine levels in milk. This method utilizes a direct greener compound for nanoparticle synthesis and melamine detection without extracting bio polyphenols from the source. Unlike previous works that employed chemical compounds unrelated to plant extracts, we emphasize the use of a greener agent for melamine detection. Our colorimetric detection method employs a one-pot sensing technique based on interference, where melamine disrupts nanoparticle formation. High melamine concentrations result in a colorless solution, while lower concentrations lead to the formation of larger, aggregated particles.

Apart from this, the role of green tea has been extensively explored as we have utilized it both as a reducing and functionalizing agent due to its remarkable antioxidant properties. This property not only helps in reducing the ions to nanoparticles but also provides them with the required stability. Green tea contains multiple antioxidants, with epigallocatechin gallate (EGCG) as its major constituent. It is rich in the –OH group, which engages multiple hydrogen bonding interactions with adulterant melamine, thereby inhibiting nanoparticle formation.

## Materials and Methods

### Chemicals

Fresh cow milk was procured from a local milk vendor. Lipton green tea was utilized for preparing the extract. Silver nitrate (99%) was purchased from Thermo Fisher Scientific, sodium borohydride was from Merck, melamine (extra pure) from Loba Chemie, trichloroacetic acid was from Qualigens, sodium hydroxide from Loba Chemie, and acidic buffer (pH 4) from Merck. Formaldehyde (37%) from Emplura, urea (extra pure) from Loba Chemie, dextrose from Merck, salicylic acid from Merck, cyanuric acid from Thermo Fisher Scientific, Whatman filter paper no. 1, 0.22 μm syringe filter, distilled water (DW), and deionized water (DI) were used. Chromic acid and aqua regia were freshly prepared and used for cleaning purpose.

### Instruments

A UV–visible spectrophotometer (Thermo Scientific GENESYS 180), an X-ray powder diffractometer (model: D8 FOCUS, make: Bruker AXS, GERMANY), a transmission electron microscope [Tecnai G2 20 S-TWIN (200 kV); resolution: 2.4A0 FEI COMPANY, USA], a field emission scanning electron microscope [JEOL], a Fourier transform electron microscope from PerkinElmer (model: SPECTRUM 100), a Zetasizer (Nano ZS90, Malvern Analytical), a weighing machine (METTLER TOLEDO ME204), a centrifuge machine (Eppendorf 5430R), an oven (Ecogian series; EQUITRON), and a magnetic stirrer (SPINOT-TARSONS) were also used during the work.

### Preparation of the Green Tea Extract

Processed green tea from the Lipton brand was utilized for the synthesis process. Initially, 50 mL of DW was heated to 100 °C in a 100 mL beaker for a duration of 15 min. Subsequently, 1 g of green tea leaves was introduced into the water and stirred using a glass rod. This tea mixture was kept aside for 15 min to allow releases of all the antioxidants and the most essential flavonoid, EGCG, into the water. The extract prepared was then subjected to double filtration using Whatman filter paper no. 1, which was then stored at 4 °C for later use.

### Preparation of Silver Nanoparticles

1 mM silver nitrate (AgNO_3_) solution was prepared by adding 0.0169 g of AgNO_3_ to 100 mL of DI water. The solution was stirred for 15 min to ensure complete dilution of the salt in the solution. Subsequently, a varied concentration of green tea extract (60, 120, and 200 μL) was introduced to AgNO_3_ solution at room temperature while maintaining a pH of 7 by addition of sodium hydroxide (NaOH). A change in color of the solution to yellow signaled nanoparticle formation.

### Pretreatment of Raw Milk

The presence of fat and protein in milk can cause potential interference with the detection mechanism, leading to erroneous results that may be either false positives or false negatives. However, this interference can be eliminated with the proper pretreatment of milk. To achieve this, fresh milk or milk spiked with various concentrations of melamine were initially treated by adding 50 mL of 10% trichloroacetic acid solution to 200 mL of milk in a 500 mL beaker. The resulting mixture was centrifuged twice, each time at 7000 rpm for 30 min, facilitating the separation of the protein and fat content. Subsequently, after successful separation of the solid casein and liquid supernatant, 6 M NaOH was introduced to the solution to adjust the solution’s pH to 7.0. The resulting supernatant was then subjected to dual filtration using Whatman no. 1 filter paper. The supernatant obtained at this stage was further refined by passing it through a 0.22 μm filter to eliminate any residual protein and fat content remaining in milk. After following this process, the resultant milk supernatant, if not spiked earlier, was then spiked with various concentrations of melamine for subsequent testing.^25^

### Colorimetric Sensing of Melamine

120 μL portion of green tea extract was combined with 400 μL of melamine-spiked milk supernatant. The resulting solution underwent stirring using a magnetic stirrer for 5 min. Subsequently, the solution’s pH was adjusted to 7 by addition of NaOH, followed by addition of 10 mL of AgNO_3_. The formation of nanoparticles and the ensuing colorimetric changes in response to the melamine’s concentration in spiked milk samples were subjected to further investigation by using a standard UV–vis spectrophotometer.

### Optimization of the Detection Process

**Figure 1:**
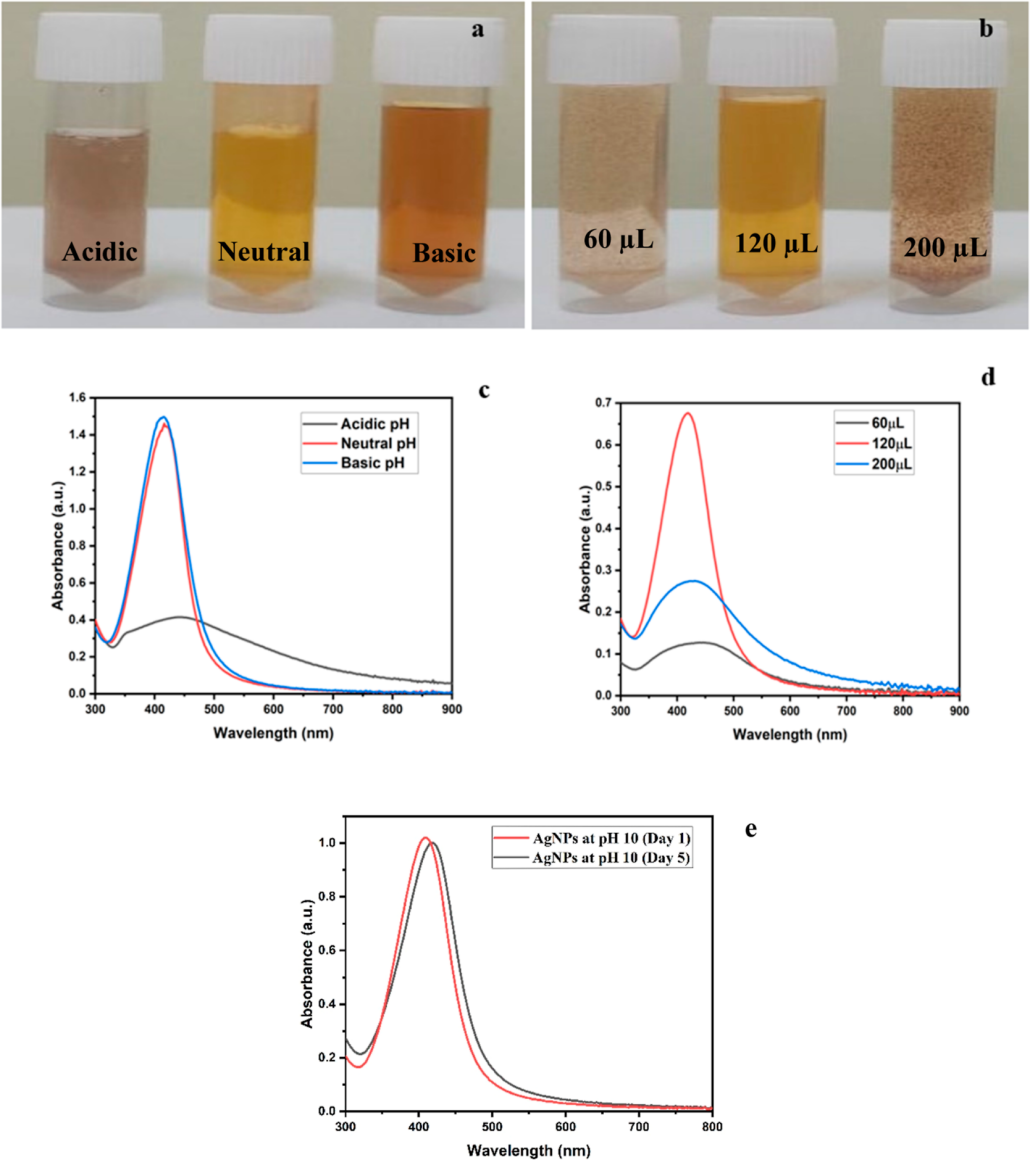
Synthesized nanoparticles at varied (a) pH conditions and (b) concentrations of reducing agent; corresponding UV–vis plots at varied (c) pH conditions and (d) concentrations of reducing agent; and (e) stability of AgNPs at a higher pH.

The pH and the quantity of reducing agent play vital roles in determining the morphology and structure of the nanoparticles. Furthermore, optimizing the synthesis process can significantly enhance the sensing performance. In the study, a pH value of 7 was found to be optimum for the detection of melamine by the green tea-functionalized nanoparticles. At acidic pH levels (pH 4), which were obtained by addition of acidic buffer during synthesis, we observed a broad UV–vis peak due to the synthesis of highly polydisperse nanoparticles. However, when the pH level was increased beyond 7 (pH 10), we observed an increased uniformity of size in the synthesized nanoparticles, which is evident from the sharp absorbance peak. However, at higher pH levels, the nanoparticles began to self-aggregate after a period of time, thereby decreasing their stability, which could potentially lead to false-positive results. The higher pH value was maintained by the dropwise addition of sodium hydroxide. Therefore, we established that a pH of 7 is the optimal condition for the detection of melamine according to this protocol as the nanoparticles remained stable even if they were stored at room temperature^26^ (Figure 1a,c,e).

Optimization was also performed by altering the amount of reducing agent, where at lower amounts, particles were not reduced properly, which resulted in the formation of aggregates. A similarly larger quantity of reducing also caused the formation of aggregates due to interaction between the reduced particles, which resulted in self-aggregation; thus, an amount of 120 μL was the appropriate amount for the reduction of ions to nanoparticles following this protocol (Figure 1b,d).

### Selectivity of the Sensing Architecture

Green tea contains multiple flavonoids and biopolyphenols, which make it a strong antioxidant. However, in the presence of melamine, it disrupts the formation of nanoparticles. To ensure the selectivity of our analysis and verify whether other potential adulterants or preservatives interfere with the sensing process, which could result in erroneous results, a comprehensive selectivity test was performed. For this assessment, milk samples were deliberately spiked with various adulterants such as formalin, urea, salicylic acid, dextrose, and cyanuric acid at a 1000 ppm concentration. The goal was to investigate whether the presence of other elements in trace amounts affects the detection process and also verify the selectivity of the sensing architecture toward the detection of melamine.

**Figure 2:**
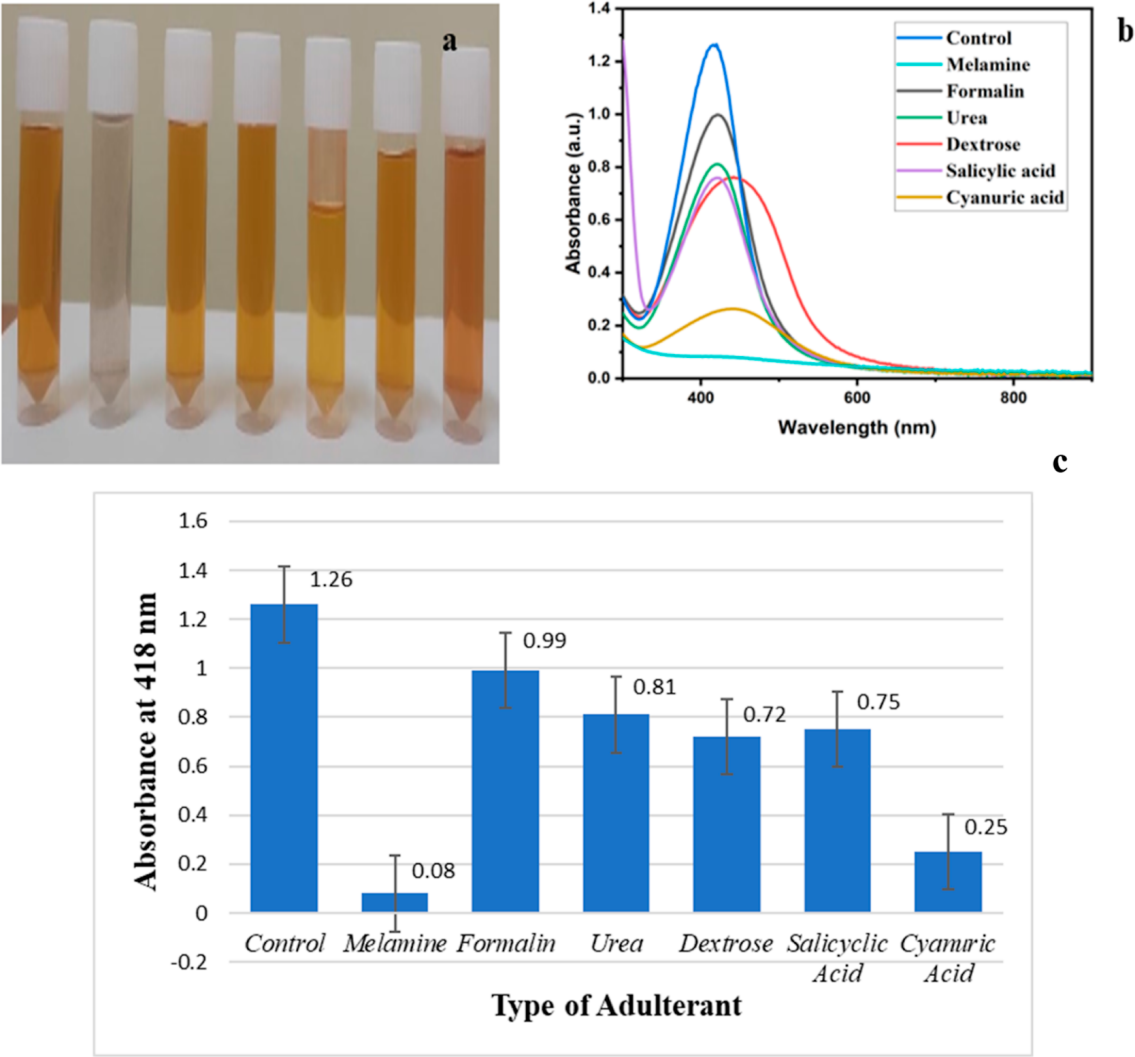
(a) Colorimetric response of nanoparticles in the presence of various adulterants, (b) UV–vis plots of nanoparticles corresponding to their colorimetric variations, and (c) histogram analysis of the selectivity test in the presence of various adulterants at a 1000 ppm concentration.

The findings indicted that none of these analytes significantly interfered with the synthesis of the nanoparticle prepared by following the protocol. The color of the salt solution changed to yellow or brown even in the presence of these adulterants. The conclusion obtained from the visual change is also supported by the UV–vis plots, which show no significant shift in the absorbance spectra when these analysts coexist in milk, except for cyanuric acid, which showed a dip in absorbance as its structure is similar to that of melamine, but there was no significant change in color of the synthesized nanoparticles. Therefore, the method is found to be appropriate for melamine detection without any observable interference from the reported adulterants (Figure 2a–c).

## Results and Discussion

### Characterizations of the Synthesized Nanoparticles

**Figure 3:**
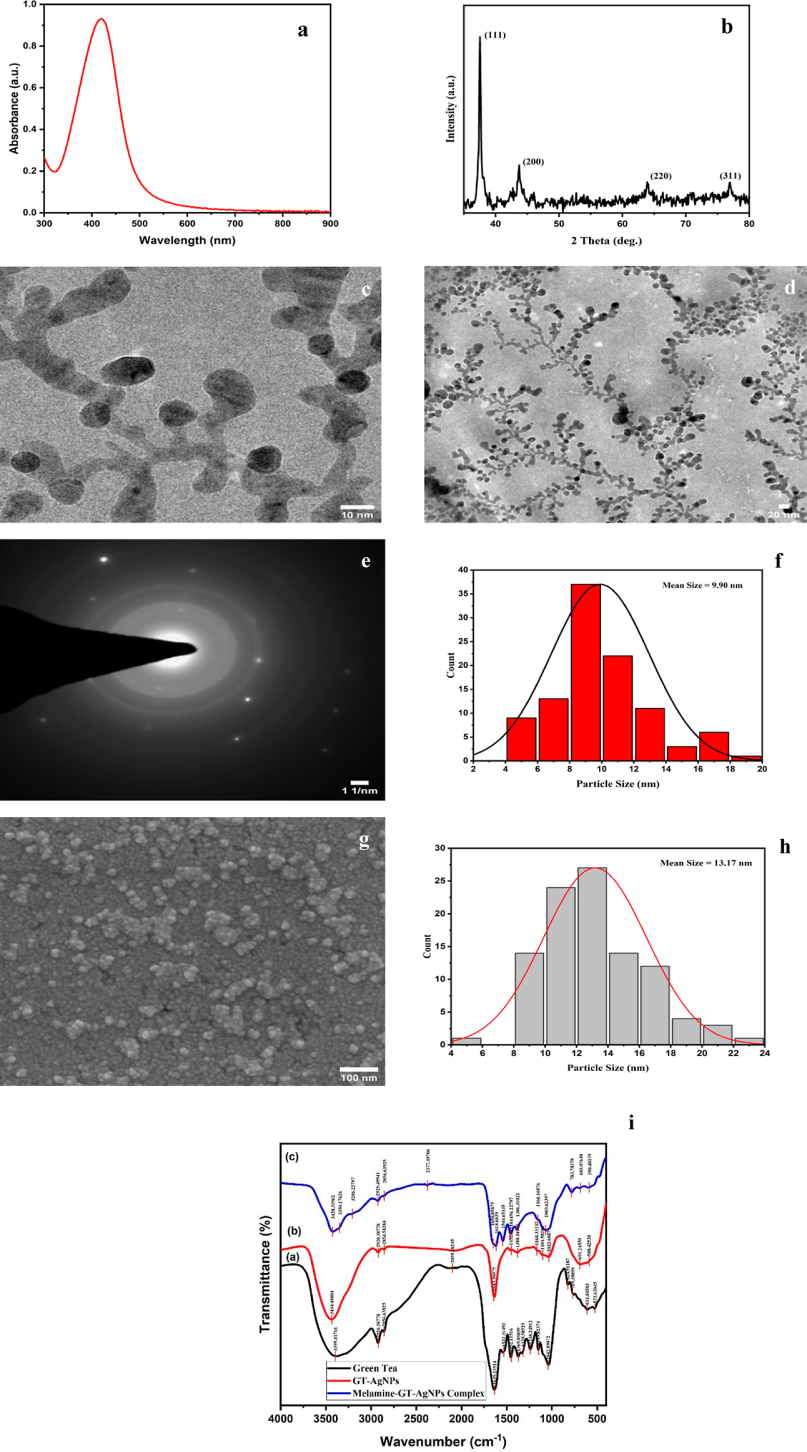
(a) UV–vis absorbance spectrum, (b) XRD pattern, (c,d) TEM images under various magnifications, (e) selected area electron diffraction image, (f) size distribution analysis from TEM, (g) FESEM image, (h) size distribution analysis from FESEM of GT-AgNPs (green tea-silver nanoparticles), and (i) FTIR spectra of green tea, GT-AgNPs, and the melamine-interacted GT-AgNPs.

To affirm the formation of nanoparticles, the synthesized nanoparticles were first subjected to UV–vis spectroscopic analysis, which confirmed the formation of silver nanoparticles. This confirmation was based on the observation of an absorbance spectrum at approximately 418 nm due to the LSPR peak of plasmonic nanoparticles (Figure 3a).^27^

Furthermore, structural characterization was accomplished through an X-ray diffraction (XRD) study, which confirmed the structural orientation of the nanoparticles to be face-centered cubic. Bragg diffraction peaks were obtained at 111, 200, 220, and 311 for 2θ values of 37.59, 43.79, 63.90, and 76.94°, respectively, with the most intense diffraction peak occurring at the (111) plane (Figure 3b). By analyzing the XRD spectrum and applying the Scherrer equation, we have determined the average crystalline size of the synthesized particles to be 11.28 nm.^28^

The Scherrer equation is given by
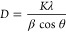
where *D* is the average crystalline size (nm), *K* is the shape factor, λ is the X-ray wavelength, β is the full width at half-maximum (fwhm) value, and θ is the Bragg angle.

Morphological parameters of the synthesized nanoparticles, such as shape and size, were elucidated through transmission electron microscopy (TEM) and field emission scanning electron microscopy (FESEM) examinations. TEM data confirmed that the synthesized nanoparticles exhibited a spherical shape with a mean diameter of 9.90 ± 3.05 nm and size ranging between 5 and 20 nm (Figure 3c–f).^29^ This was further corroborated by FESEM images, which also depicted spherical nanoparticles possessing an average size of 13.17 nm (Figure 3g,h).^30^ The nanoparticle size is observed to be comparable to the crystalline size of the nanoparticles calculated by using the Scherer equation.

The Fourier transform infrared spectroscopy (FTIR) method was employed for the comprehensive characterization of nanoparticles, specifically focusing on identifying the functional groups responsible for the reduction and encapsulation of nanoparticles. In the IR spectra of green tea, a notable broad band dip at 3395 cm^–1^ indicated the H-bonded OH stretch. Additional features included transmittance dips at 2920 and 2856 cm^–1^, corresponding to the C–H stretch in alkanes and the O–H stretch in carboxylic groups, respectively. A distinct dip at 1635 cm^–1^ was attributed to the C=O stretch in polyphenols and the C=C stretch in aromatic rings. Further dips at 1369, 1320, 1238, and 1149 cm^–1^ provided insights into the presence of carboxylic groups, aromatic nitro compounds, C≡C, and the secondary alcohol C–O stretch, respectively. The identification of C–O stretching in amino acids manifested as a dip at 1042 cm^–1^. A weak transmittance dip at 824 cm^–1^ was associated with C–H out-of-plane bending, while peaks at 769, 611, and 522 cm^–1^ indicated the presence of aliphatic chloro and bromo compounds, signifying C–Cl and C–Br stretches. Comparing these results with those obtained for the green tea extract and the corresponding reduced nanoparticles, significant dips were observed at positions 3434, 2920, and 2854 cm^–1^, indicative of the H-bonded OH stretch, C–H stretch in alkenes, and O–H stretch caused by carboxylic acid, respectively. A highly intense transmittance dip at 1643 cm^–1^ was attributed to the C=O stretch in polyphenols and the C=C stretch in aromatic rings, both present in green tea and thus contributing to the functionalization of the nanoparticles. Additionally, a transmittance dip at 1369 cm^–1^ affirmed the presence of carboxylic groups, collectively confirming the proper functionalization of the nanoparticles and the reduction of metals through the presence of these functional groups (Figure 3i).^31,32^

### Mechanism of the Sensing

**Figure 4:**
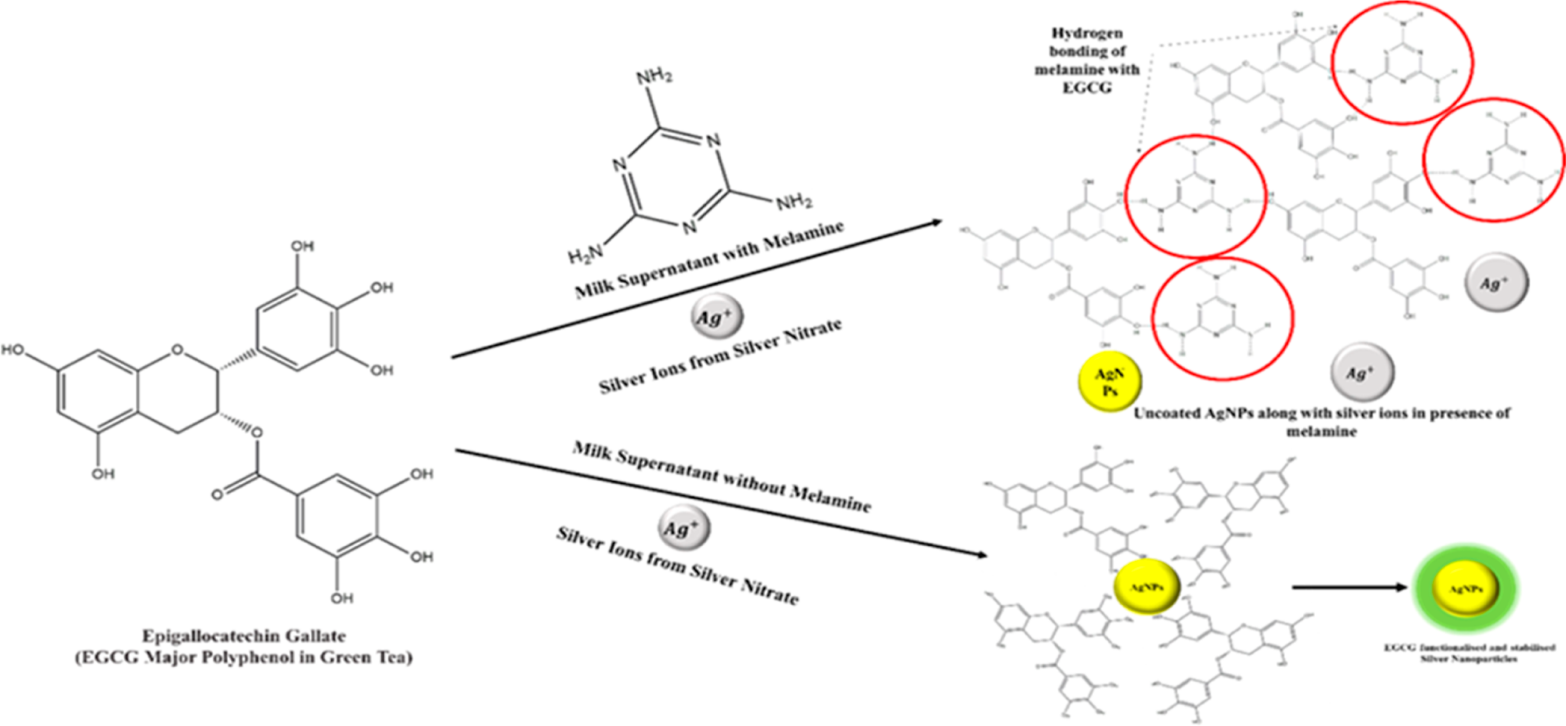
Schematic illustration demonstrating the mechanism of sensing melamine by green tea-functionalized silver nanoparticles.

**Figure 5:**
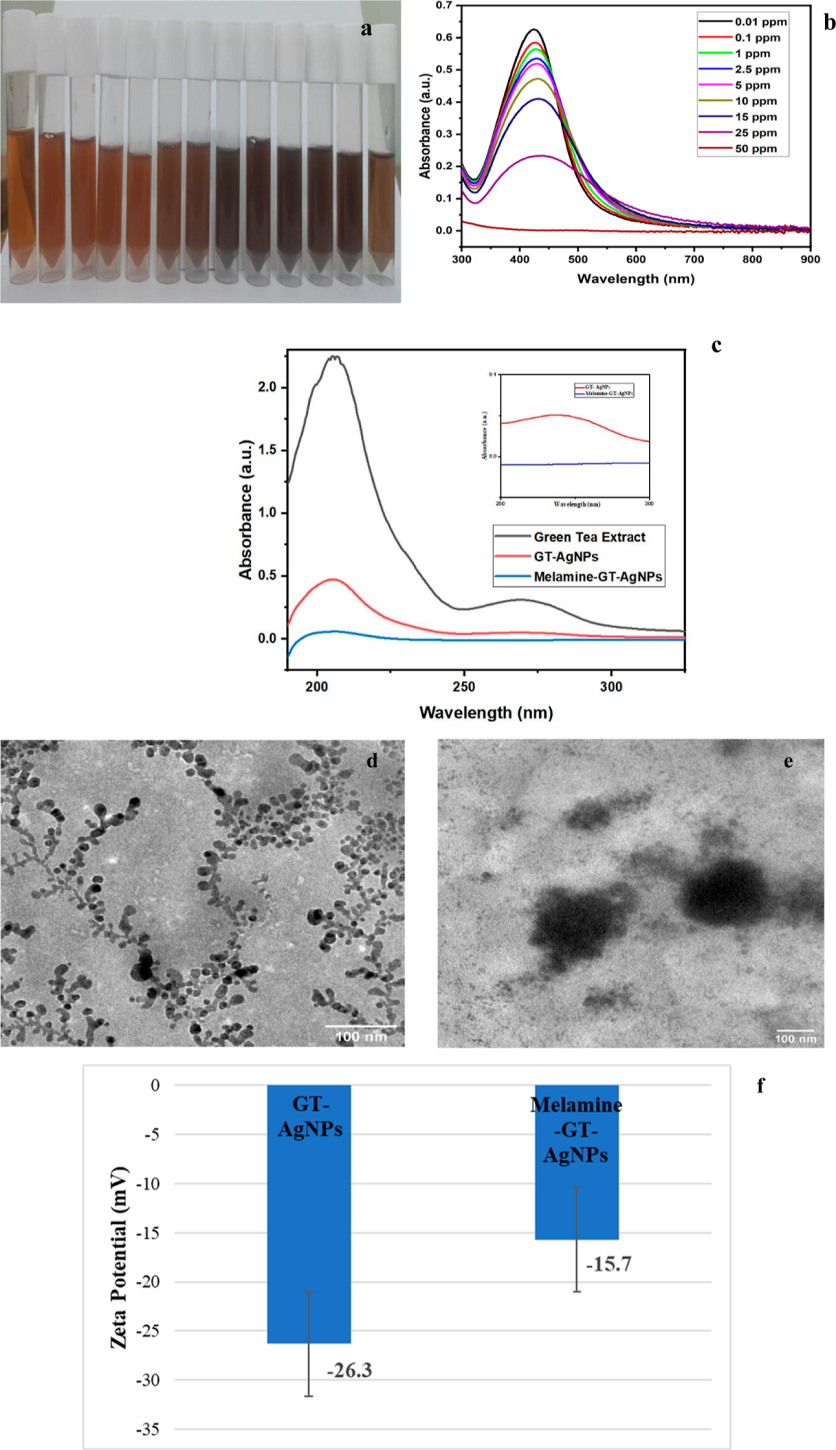
(a) Pictorial representation of the colorimetric change of GT-AgNPs under various concentrations of melamine; (b) UV–vis plot of the interference-based synthesized GT-AgNPs in the presence of melamine at varied concentrations; (c) UV–vis spectra of green tea extract, green tea AgNPs, and melamine interaction with nanoparticles reduced by green tea; (d) TEM image of GT-AgNPs in the absence of melamine; (e) TEM images of GT-AGNPs in the presence of melamine; and (f) zeta potential values of GT-AgNPs and the melamine–GT-AgNP complex.

This sensing protocol employs an eco-friendly element, green tea, which not only serves as a reducing agent by converting the metal ion (Ag^+^) to nanoparticles but also plays a pivotal role in detecting melamine. Green tea being an eco-friendly element mitigates any adverse environmental impacts. In the study, we have exploited green tea not only as a green reducing agent but also as a sensing element toward one-pot interference-based detection of melamine. The phytochemicals present in green tea are responsible for reducing the ions to nanoparticles but do not interfere with milk supernatant. This is due to the removal of interfering proteins and fats from the milk during a preprocessing step. In comparison with the previous reported works, where detection of melamine was performed by first nanoparticle synthesis followed by its complex functionalization for selective colorimetric sensing, our sensing approach takes a distinctive path (Figures 4 and 5a,b).^26,33^

Green tea contains a diverse category of catechins, polyphenols responsible for melamine detection. These include (+)-catechin, (−)-epicatechin (EC), (+)-gallocatechin (GC), (−)-epicatechin gallate (ECG), (−)-epigallocatechin (EGC), and (−)-EGCG. The presence of various catechins was confirmed through UV–vis absorbance analysis of the green tea samples, revealing a distinct peak around 271 nm. According to the literature, this peak aligns with the absorbance peaks of EGCG and ECG. The peak obtained at 273 nm suggests a mixture of catechins, primarily EGCG, as it is the dominant polyphenol in the green tea extract.^34^ The peak was evident in the nanoparticles prepared with the extract, signifying the effective functionalization and reduction of the nanoparticles by the extract. However, in the presence of the analyte, plentiful polyphenol interacts with melamine, resulting in the formation of a novel compound that lacks any observable peak at that position. This implies that the extract can serve as an active agent for determining melamine in nanoparticles (Figure 5c).

The analyte and green reducing agent interact via hydrogen bonding. EGCG, being the abundant polyphenol and antioxidant present in green tea, reduces the ions into nanoparticles. However, in the presence of melamine, which is a compound containing three –NH_2_ groups, it interacts with the –OH group of EGCG via hydrogen bonding. This interaction inhibits the formation of nanoparticles as there are fewer available active sites for reducing the ions to particles.^35,36^ The accuracy of the results was confirmed through triplicate experiments using melamine-spiked milk samples. Consistent findings were observed in all cases, demonstrating a uniform limit of detection (LOD) and high repeatability. TEM images of nanoparticles also confirmed the sensing scheme, where, in the absence of melamine, uniform scattering of nanoparticles can be observed, with each encapsulated by polysaccharides and bound with polyphenols. In the presence of melamine, the particles no longer remain in the nanoregime, and there is no observable encapsulation. This suggests that polyphenols interacted with melamine instead of the silver ions present in the solution, which resulted in formation of larger aggregates (Figure 5d,e).

In continuation of this investigation, an FTIR study was conducted to understand the interaction between melamine and green tea extract, influencing the formation of nanoparticles. The FTIR spectra of the complex revealed a new transmittance peak at 2377 cm^–1^, attributed to the presence of C≡C stretching. Additionally, a subtle dip in transmittance at 1650 cm^–1^ indicated the existence of an amide group. The interaction between polyphenols and the amide group was manifested in broadening of the graph, as evident in the FTIR spectra. Three distinct transmittance dips were observed at 3428, 3350, and 3206 cm^–1^, corresponding to the polymeric stretch of the OH group. These features underscored the interaction between melamine and the polyphenolic components of green tea, providing insights into the inhibitory effect on nanoparticle formation (Figure 3i).^31^

The zeta potential findings were consistent with the obtained results, aligning with other observations. Green tea-functionalized silver nanoparticles exhibited a higher zeta potential (−26.3 mV) than that of the complex formed by integrating melamine with green tea-AgNPs (−15.7 mV). A decrease in the zeta potential signals a reduction in the surface charge, leading to particle aggregation. Due to a smaller amount of electrostatic interaction between the particles, the introduction of melamine in milk samples diminished particle stability, fostering agglomeration and subsequently reducing the zeta potential value.^37^

In the presence of melamine, the reducing agent engages with it rather than the metal ions, resulting in the generation of fewer nanoparticles or aggregated nanoparticles of larger size with altered shapes. This phenomenon results in colorimetric changes in the synthesized nanoparticles, which could be correlated with the melamine’s concentrations in spiked milk. Melamine is a strong nucleophile containing six hydrogen bonds, which enable strong interactions with the reducing agent, making it an excellent onsite sensor.^38^ However, the nanoparticles lose their ability for reuse in sensing once the initial sensing is conducted as they tend to aggregate in the presence of melamine. Subsequent sensing attempts with the same set of nanoparticles are thereby impeded.

### Sensor Parameters

To determine the LOD, we plotted a calibration graph correlating absorbance with the concentration of melamine. The colorimetric changes observed with increments in the concentration of melamine were analyzed by a UV–vis spectrophotometer, which revealed a substantial shift and decrease in absorbance. This can be attributed to a decrease in the number of nanoparticles or the formation of larger-sized aggregated particles in the presence of melamine. Based on the response obtained from the UV–vis spectrum, a calibration graph was plotted to determine the LOD of the interference sensor. The LOD of the sensing scheme was calculated by using the slope (*s*) and the standard error (σ) of the calibrated graph using the formula
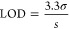
1

**Figure 6:**
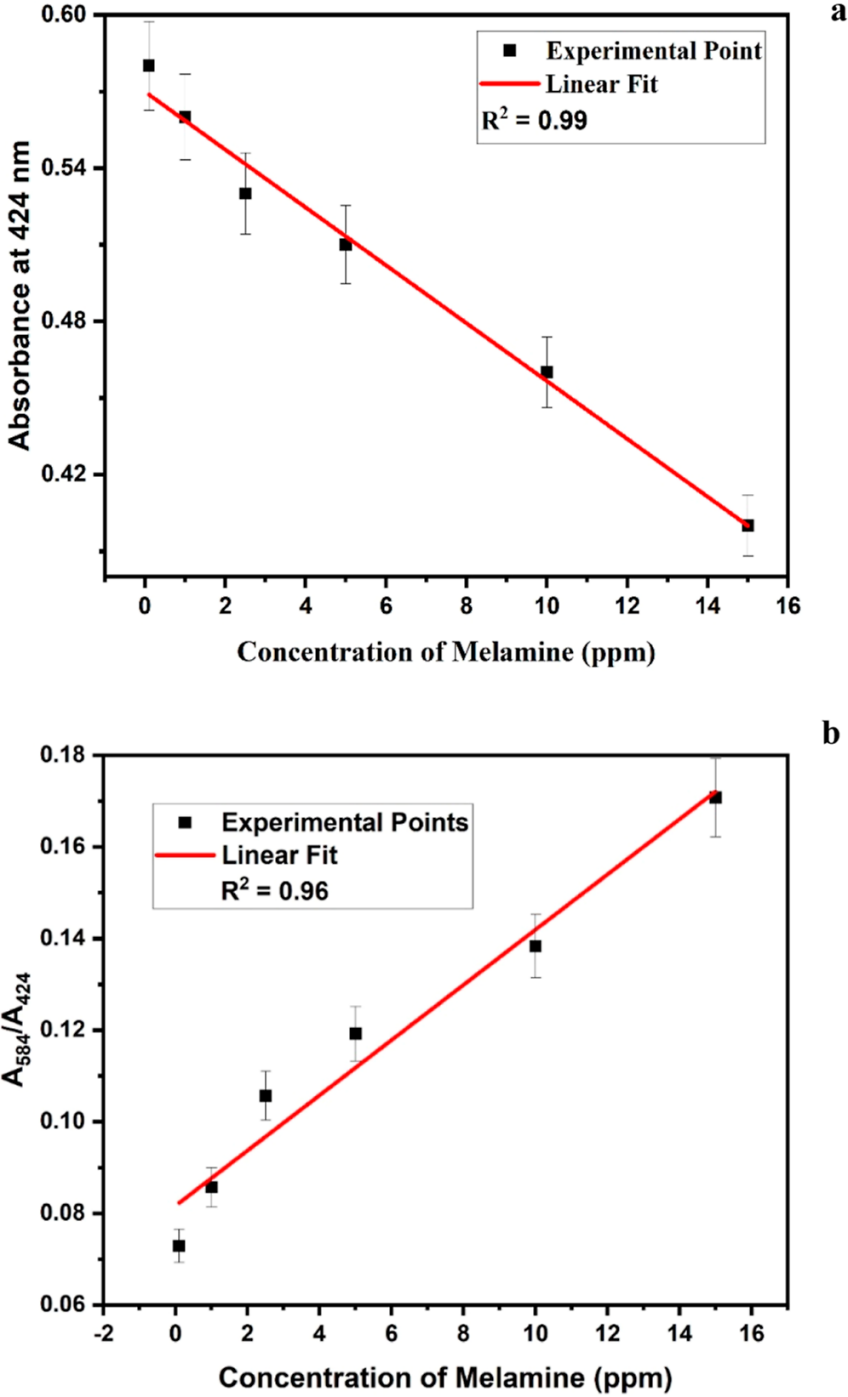
Linear calibrated graph between (a) absorbance and the concentration of melamine in milk and the (b) absorbance ratio and the concentration of melamine in milk.

We also assessed the recovery rate, which was found to be 93% by comparing the theoretically expected amount of melamine added to milk with the actual amount of melamine present in milk.
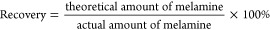
2By analyzing all the results, the LOD of the sensor was found to be 1.44 ppm, much lower than the permissible limit suggested by FSSAI, which is 2.5 ppm (Figure 6a).^12,13^

The sensitivity and dynamic range of sensing are also calculated from the calibrated graph. Sensitivity is determined by using the slope of the graph, yielding a value of 0.01 au for a unit change in ppm. This implies that a 0.01 au change in nanoparticle absorbance is observed for every 1 ppm alteration in the melamine concentration in milk. This sensitivity is achieved within a dynamic range spanning 0.1 to 15 ppm. Within this range, the sensor demonstrates a linear response in absorbance corresponding to changes in the melamine concentration in milk (Figure 6).^39^

Another linear calibrated graph was plotted between the absorbance ratio and the concentration of melamine in milk. It was observed that as the concentration of melamine in milk increased, the absorbance of nanoparticles not only decreased at 424 nm but also increased at 584 nm due to the aggregation of nanoparticles in the system due to the presence of melamine (Figure 5b).

### Analytical Validation by High-Performance Liquid Chromatography

**Figure 7:**
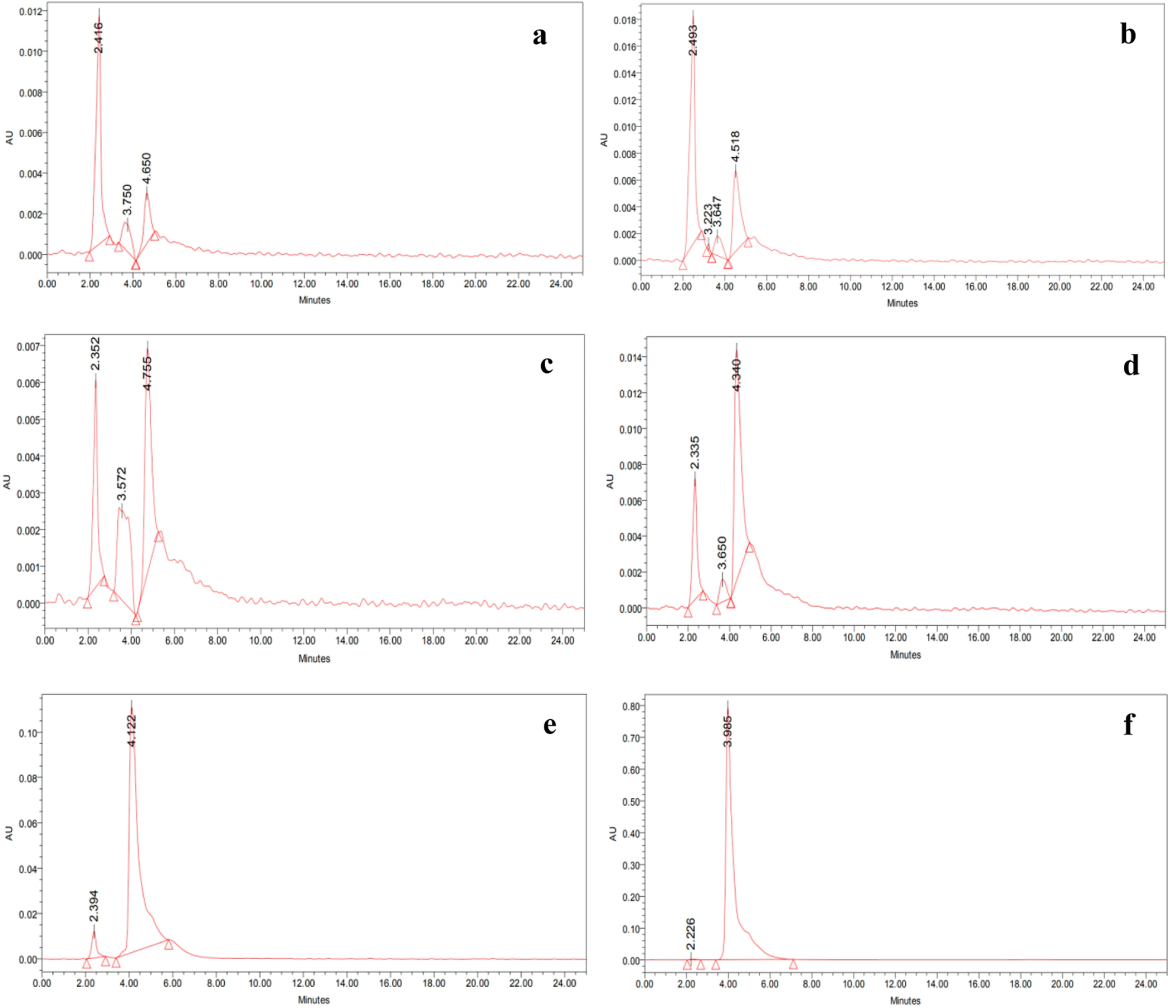
HPLC data of melamine in milk at various concentrations: (a) 0, (b) 0.1, (c) 1, (d) 10, (e) 100, and (f) 1000 ppm.

To validate the results obtained by this sensor for detecting melamine in raw, preprocessed milk, we employed high-performance liquid chromatography (HPLC) as a validation technique. In this regard, we conducted HPLC analysis of some of the melamine-spiked milk samples, following a methodology.^35^ For this, a standard solution was utilized, which is 1000 ppm melamine dissolved in water. A C18 column was used for separation with a UV detector (240 nm). To perform the analysis, a 3 μL volume of the sample was injected into the column, where the mobile phase consisted of acetonitrile and water and the rate of flow was 0.7 mL/min. A significant peak emerged at a retention time of approximately around 4 min, corresponding to that of the presence of melamine in the milk samples. A consistent increase in the peak could be observed with an increment in the concentration of melamine in spiked milk samples. This technique validated the results obtained from the UV–vis spectroscopic analysis (Figure 7).

### Comparative Analysis

A comparative study has been conducted to analyze the sensing performance of this sensing scheme against the melamine detection schemes that are already reported in the literature (Table 1).

**Table 1:** Comparative Analysis Study

detection type	reducing agent	functionalizing agent	selectivity	recovery	LOD/lowest limit	validation	reference
colorimetric (AuNPs)	trisodium citrate	trisodium citrate	interfering ions Ca^2+^, K^+^, Na^+^, NH^4+^, Cl^–^, SO_4_^2–^, and PO_4_^3–^	not performed	1 ppm	not performed	Guo et al., 2010^25^
colorimetric (AuNPs)	trisodium citrate	1-(2-mercaptoethyl)-1,3,5-triazinane-2,4,6-trione	cytosine, uracil, and thymine	not performed	2.5 ppb	not performed	Ai et al., 2009^40^
colorimetric (AgNPs)	sodium borohydride	trisodium citrate	vitamin C, lactose, glucose, polypeptide, NH^4+^, Na^+^, K^+^, Ca^2+^, SO_4_^2–^, Cl–, NO^3–^, and Cl^–^	88.83–114.29%	2.32 μM	not performed	Ping et al., 2012^41^
colorimetric interreference based (AgNPs)	dopamine	dopamine	phenylalanine, dl-leucine, l-glutamate, sulfanilic acid, Mg^2+^, galactose, glucose, lysine, urea, and cysteine	98.5%	0.01 ppm	not performed	Ma et al., 2011^8^
colorimetric (AgNPs)	sodium borohydride	sulfanilic acid	lysine, tryptophan, methionine, leucine, isoleucine, phenylalanine, valine, NO^3–^, pyrophosphate, citrate, CO_3_^2–^, EDTA, Ca^2+^, Mg^2+^, Zn^2+^, Fe^3+^, Na^+^, glucose, fructose, and sucrose	103–109%	10.6 nM	not performed	Song et al., 2015^20^
colorimetric interreference based (AgNPs)	ascorbic acid	ascorbic acid	dextrose, glycine, leucine, citric acid, lactose, zinc, and sodium	not performed	0.1 ppm	by HPLC	Varun et al., 2017^35^
surface enhanced Raman scattering (AuNPs)	trisodium citrate	trisodium citrate	not performed	96–100%	0.17 mg L^–1^	not performed	Giovannozzi et al., 2014^42^
LSPR-based with optical fibers (AuNPs)	trisodium citrate	trisodium citrate	glucose, fructose, lactose, and ammeline	99.2–111%	33 nM	not performed	Chang et al., 2017^43^
LSPR-based cuvette cell (AuNPs)	trisodium citrate	*p*-nitroaniline	cyanuric acid, uracil, urea, and *m*-phenylenediamine	not performed	0.01 ppb	not performed	Oh et al., 2019^44^
device based (AgNPs)	sodium borohydride	trisodium citrate	not performed	not performed	not performed	not performed	Ramalingam et al., 2017^45^
interference-based colorimetric (AgNPs)	green tea	green tea	urea, formalin, salicylic acid, dextrose, and cyanuric acid	93%	1.44 ppm	by HPLC	reported work

## Conclusions

This work reports a simple approach for detecting a highly toxic milk adulterant, melamine, following a less explored route, which is interference-based colorimetric sensing. In the reported work, green tea has a dual role as it acts both as a reducing agent for nanoparticle synthesis and as a key component for milk adulterant sensing. The colorimetric change observed results from the interaction between the analyte and the reducing agent, leading to inhibition of nanoparticle formation or the creation of larger-sized aggregates. In this regard, the fabricated sensing scheme is simple, enabling one-pot synthesis and detection in a single step. The schemes offer a low detection limit of 1.44 ppm and a recovery value of 93% with a dynamic range of sensing between 0.1 and 15 ppm. To validate our results, we conducted HPLC analysis, which served as an additional layer of confirmation for our sensing scheme’s efficacy.

## Data Availability

All the data set related to the research work performed has already been added to the manuscript.

## Supporting Information Available

Colorimetric detection of melamine in aqueous media (PDF)
