# Supplementary material for: One-Pot Interference-Based Colorimetric Detection of Melamine in Raw Milk via Green Tea-Modified Silver Nanostructures
Source: ACS Omega. 2024 May 9;9(20):21879–90. doi: 10.1021/acsomega.3c09516 (PMC11112553; doi:10.1021/acsomega.3c09516)
Supplement: Supplementary file 1 — ao3c09516_si_001.pdf [file ao3c09516_si_001.pdf]

Supporting Information

One-pot Interference-Based Colorimetric Detection of Melamine in raw milk *via* Green Tea modified silver nanostructures

Upama Das[1], Rajib Biswas[1*], Nirmal Mazumder[2]*

[1]*Applied Optics and Photonics Research Laboratory, Department of Physics, Tezpur University, Assam*

2 Department of Biophysics, Manipal School of Life Sciences, Manipal Academy of Higher Education, Manipal, Karnataka, India-576104

*Corresponding Author: rajib@tezu.ernet.in, nirmal.mazumder@manipal.edu

Colorimetric detection of melamine in aqueous media: Distilled water

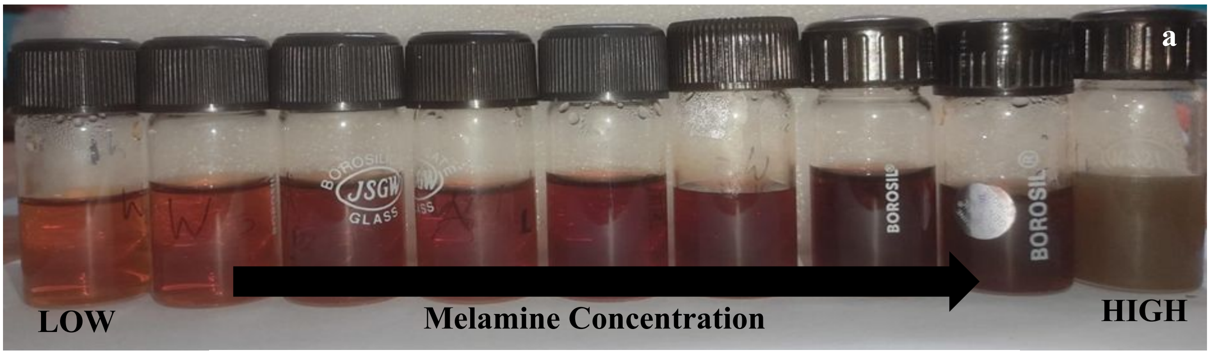

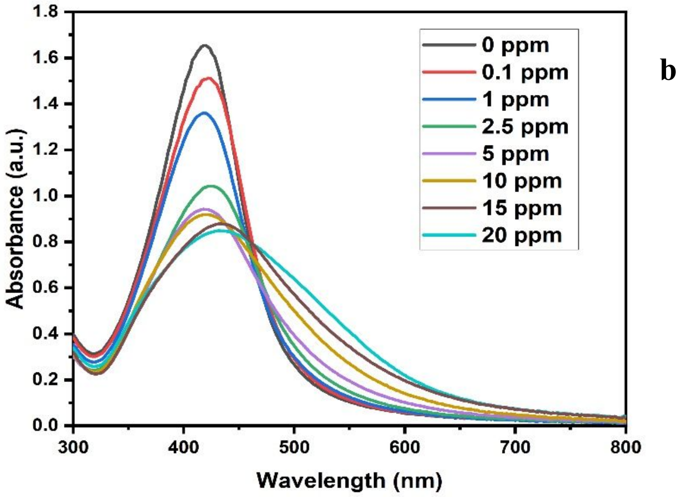

Figure S1.: (a) Pictorial representation of colorimetric change of GT-AgNPs under various concentration of melamine in water, (b) UV-vis plot of the interference based synthesised GT-AgNPs in presence of melamine in water at varied concentration.

a

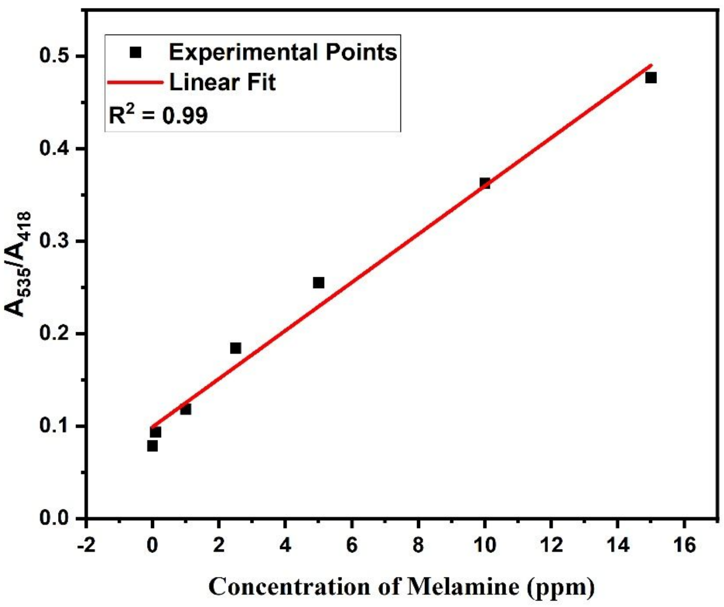

b

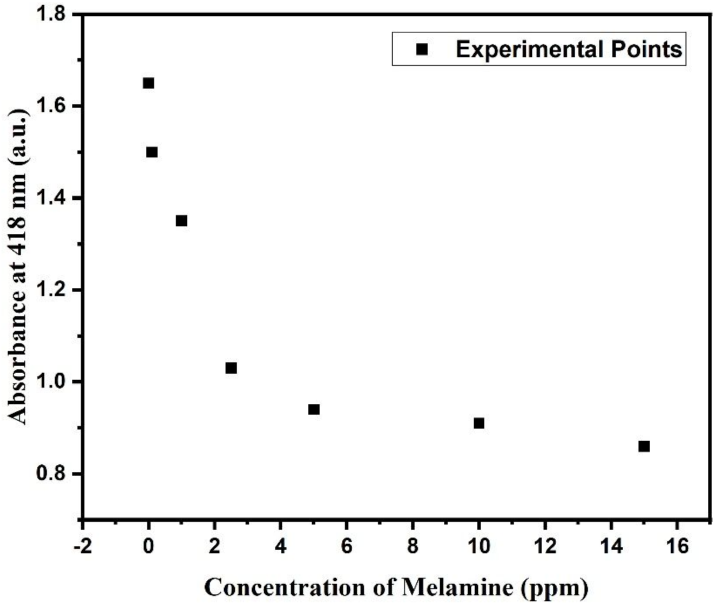

Figure S2.: Linear calibrated graph between (a) absorbance ratio and concentration of melamine in water, and (b) absorbance and concentration of melamine in water.

In this study, we conducted experiments to assess the efficacy of the detection scheme in aqueous media, specifically water. Our investigation revealed a direct correlation between the absorbance ratio and the concentration of melamine in water, with a linear increase as the melamine concentration rose. In contrast, the absorbance in relation to melamine concentration exhibited an exponential decrease. Utilizing the linearly calibrated graph, we determined a Limit of Detection (LOD) of 1.22 ppm, a value significantly below the permissible limit.

Notably, the colorimetric sensor demonstrated its functionality within a dynamic range of 0.1 ppm to 15 ppm in water, exhibiting a sensitivity of 0.02 change in absorbance ratio per unit change in melamine concentration. This underscores the sensor's capability to operate effectively and detect melamine concentrations in water within a wide and practically relevant range.
